# Investigation of joint formation in aluminum wire and nickel-coated copper terminals using ultrasonic welding

**DOI:** 10.1038/s41598-025-08477-2

**Published:** 2025-07-01

**Authors:** Haohan Zhang, Lun Zhao, Zeshan Abbas, Wanlu Hong

**Affiliations:** 1 School of Mechanical and Electrical Engineering, Yunnan Open University, Kunming, 650500 China; 2 Tech X Academy, Shenzhen Polytechnic University, Shenzhen, 518055 China

**Keywords:** Ultrasonic welding, Nickel-coated copper terminals, Intermetallic compound, Microstructures, Failure analysis, Engineering, Mechanical engineering

## Abstract

The presence of intermetallic compounds (IMC) can significantly degrade the mechanical properties of joints, leading to premature failure. This paper presents a detailed investigation into the mechanical manufacturing and forming quality of ultrasonically welded joints between aluminum wire, copper terminals and nickel-coated copper terminals. The electroplated nickel coating enhances the corrosion resistance and operating temperature tolerance of copper substrate. The changes in failure load and energy absorption of welded joints were analyzed by examining the force-displacement curves. Microscopic morphology and elemental composition of joints were studied using scanning electron microscopy (SEM) and energy dispersive spectrometry (EDS). The nickel-coated copper terminals inhibit metallurgical bonding to some extent and reduce the formation of IMCs at the weld interface. The average failure load of the Al-Ni joint was 2558.85 N which is 2.05% lower than Al-Cu joint, which had an average failure load of 2612.52 N. The average energy absorption of the Ni joints (21.65 J) was 20.14% lower than Al-Cu joints (27.11 J). The results indicate that while the coating has a negligible effect on joint strength, it does weaken the impact and seismic resistance of the joint. The joints can be used in manufacturing electrical components such as connectors, switches and sensors, where reliable electrical conductivity and resistance to corrosion are critical.

## Introduction

Aluminum wires with excellent corrosion resistance, light weight, cheaper value and adhesion are used in automobile manufacturing industry compared to copper wires^1^. Lithium battery packs consist of many single lithium batteries. Batteries are connected in series with common connectors and materials for lithium battery tabs are mainly copper and aluminum^2^. A large amount of high-quality Al/Cu heterogeneous junctions is more suitable for production of connecting junctions in green electric vehicles and ensures maximum power supply efficiency. Coated surfaces can reduce effect of IMC formation, reduce effect of corrosion and prevent direct contact of dissimilar metals^3,4^. Advantage derives from the fact of coated surfaces with nickel-coated copper terminals. It can effectively block direct contact of oxygen and water molecules with wires and reduces risk of copper oxidation and avoids formation of copper green color^5,6^.

So far, commonly used welding methods for connecting lithium battery tabs and producing smooth power transmission for green vehicles include laser welding, soldering and resistance welding, etc. However, resistance soldering of the copper/aluminum joint has poor performance due to its resistance and conductivity^7^. Although laser welding is currently used to obtain higher quality dissimilar copper/aluminum joint, but a thicker intermediate phase will be generated at the weld interface^8^. The intermetallic compound (IMC) and resistance of IMC is obviously higher than base material, which will greatly increase tab current of lithium batteries. Hence, the greatest loss of energy occurs during battery use. This affects the lithium battery and promotion of pool groups due to creation of resistance. The traditional welding method cannot play a good role because of great differences in physical properties of dissimilar metals^9^. Ultrasonic welding can realize strong connection between Al wire harness and coated terminal, which is widely used in production of automotive wire harness, electronic industry, communication equipment and photovoltaic industry^10^. Ultrasonic welding of coated copper terminal and Al wire has always been difficulties of industry, which is due to high melting point of Cu and low melting point of Al^11,12^. High local temperature can lead to Al wire melting into liquid phase when high temperatures are encountered, affecting coated surfaces. Welded joints tend to experience different forms of failure due to complexity of environmental factors and static performance parameters during welding^13^. It can have a significant impact on microstructure morphologies, mechanical properties, corrosion resistance and physical characteristics of welded joint^14^. The high-temperature tensile properties of dissimilar welded joint were investigated and clarified^15,16^. Dewang et al. introduced the ultrasonic welding of magnesium-titanium dissimilar metals. The study investigated influence of welding parameters on mechanical property by experimentation and artificial neural network^17^. Dhara et al. introduced the impact of ultrasonic welding on multi-layered Al–Cu joint for electric vehicle battery applications^13^. Fuxing et al. presented joints formation and bonding mechanism of ultrasonic welded multi-strand single core copper cables with copper terminals^5^.

To gain a deeper understanding of the potential influence of coated surfaces on the performance of Al wire/Cu soldered joints under optimized welding parameters, this study employs 25 mm² aluminum wire, copper terminals and nickel-coated copper terminals to conduct a series of ultrasonic welding tests under consistent parameters. The tests focus on the analysis of joint strength, microscopy of the joints and failure modes. The mechanical properties and morphological characteristics of the welded joints are thoroughly examined.

## Experimental procedure

**Fig. 1:**
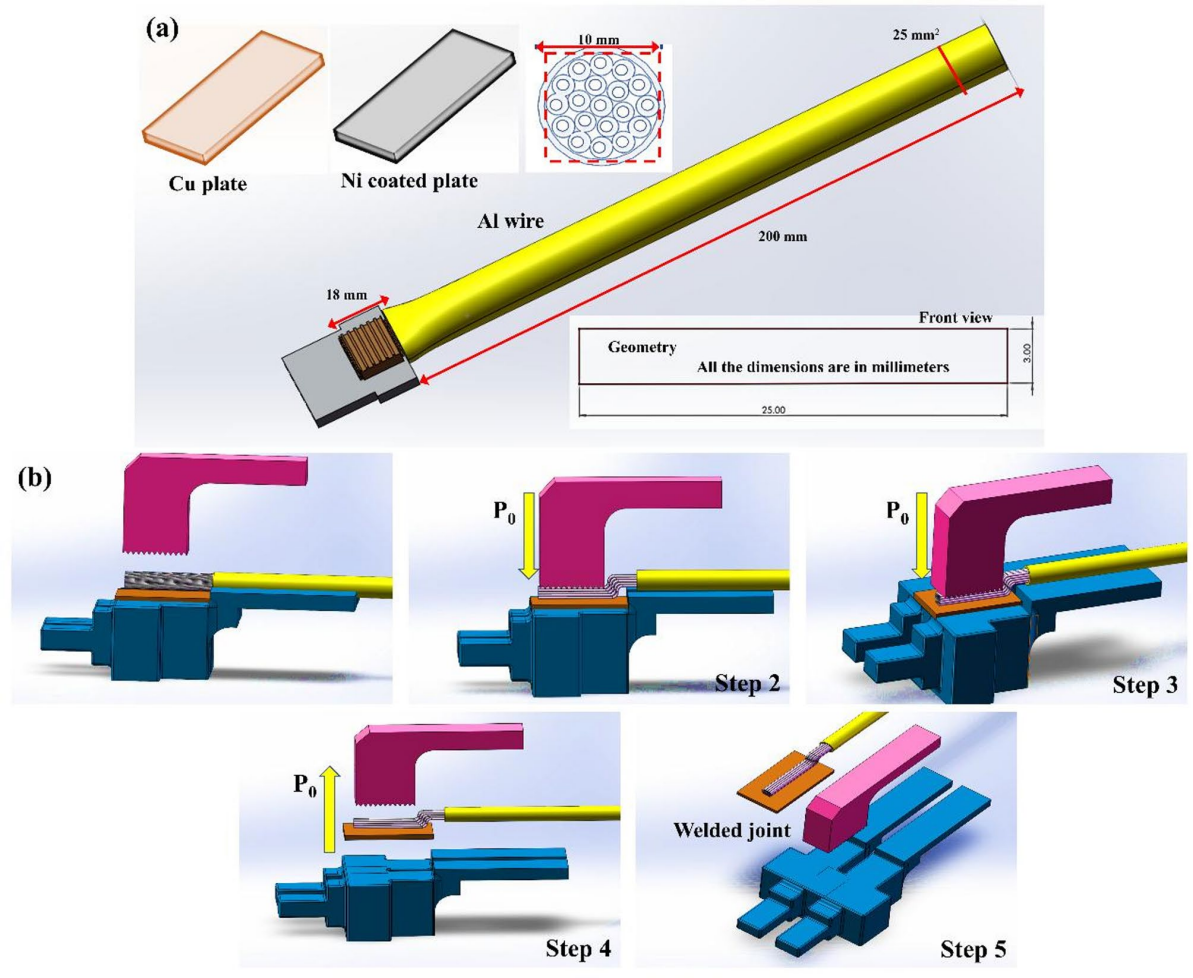
(**a**) Diagram of USW materials dimensions and (**b**) Steps of USW process.

Figure 1a,b presents the dimensions of the selected materials and the process steps involved in ultrasonic welding (USW). Ultrasonic welding is a solid-state welding process where high-frequency ultrasonic vibrations are applied to the workpieces. The process begins with the transfer of energy from the weld head (also known as the sonotrode) to workpiece. The direction of vibration is parallel to the surface of the weldment, allowing the ultrasonic waves to create localized heating at the interface between the materials. It results in the formation of a strong and metallurgical bond without the need for external heat sources. The energy is concentrated at the weld interface and facilitated the bond formation under applied pressure which completes the USW process.

This research work selects welding parameters based on the shape of material, thickness and shape of the weldments to standardize the process and facilitate the analysis of joints with different combinations. In this study, 25 mm² aluminum wire and copper terminals were chosen as the materials for the experiment. The aluminum wire had a length of 200 mm and a cross-sectional area of 1.8 mm². The copper terminals were 45 mm in length, 25 mm in width and 3 mm in thickness. Additionally, nickel in length 25 mm and 3 mm in thickness. Moreover, nickel-coated copper terminals could also be used in similar setups for improved corrosion resistance and joint quality. Figure 1a shows the dimensions of these materials used in the study which are carefully selected to ensure compatibility with the ultrasonic welding process. The ultrasonic welding machine used in this study has a maximum power output of 13 kW with an operating frequency of 20.11 kHz. The high-frequency vibrations of machine are essential for the successful welding of materials. The process parameters such as welding time, amplitude, welding pressure and production efficiency are crucial to ensure the success of welding process. Welding time was varied from 0.005 to 9.999 s, while the welding amplitude was set between 6 and 10 μm which is the distance of sonotrode (weld head) moves during each vibration cycle. The values were selected to optimize the bond formation and minimize material distortion. The welding pressure applied during the ultrasonic welding process is crucial for the success of bond. The welding pressure was set to 180 kPa which ensures sufficient contact between the materials without causing excessive deformation or overheating. The ultrasonic vibrations generate localized heat at the interface, ensuring a strong and durable bond between the aluminum wire and copper terminal. The vibration generated by welding head is parallel to the surface of workpieces which is why it is referred to as a shear-wave process. The vibrations cause atomic-scale movement at the interface of the materials and allows them to bond under pressure without melting. The welding energy supplied during the process was set to 1600 J which is sufficient to overcome the material’s resistance and form a solid bond. Energy is transferred from the weld head to workpiece. This localized heat softens the materials and allows for a strong metallurgical bond. Welding parameters were optimized to achieve the best joint quality. The optimized parametric selection for the experiments was chosen. The welding pressure was set at 180 kPa to ensure adequate contact pressure during the process. The welding amplitude was set at 46 μm to achieve the desired level of vibration and energy transfer at the material interface. Thus, the welding energy was applied 1600 J which provides enough power to overcome the initial resistance between the materials, ensuring that the joint forms properly. The frequency of the ultrasonic waves was maintained at 20.11 kHz and a typical frequency for such welding operations provides efficient energy transfer and precise control of the welding process. Figure 1b shows the process flow and dimensions of the weld area with the welding head having an area of 7.8 mm × 5.2 mm and a tooth depth of 1.2 mm. The dimensions ensure that the ultrasonic vibrations focus on the right area for bonding and create a small but strong weld joint. After the welding process, the joint quality is evaluated through microscopic examination to check for failure modes and morphological characteristics of the welded joint. The parameters are further optimized based on these evaluations to achieve a reliable and durable bond. The production efficiency of the welding machine is also an important consideration. The efficiency was found to be between 120 pcs/H and 150 pcs/H, indicating the speed at which the machine can complete the welding process for a given number of parts per hour. This is useful for scaling up production while maintaining high quality.

## Results and discussion

### Static properties of welds

**Fig. 2:**
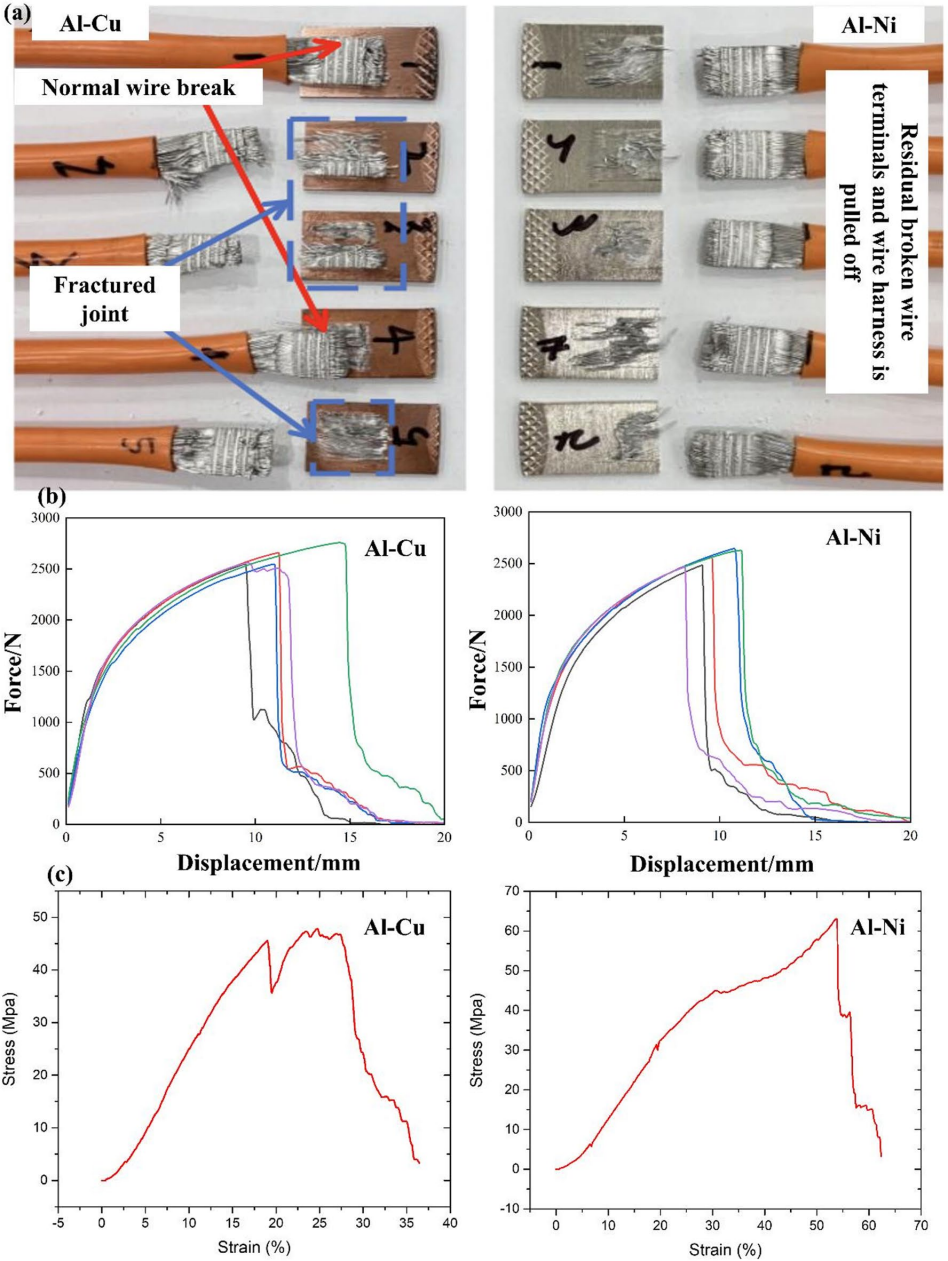
(**a**) USW welded samples; (**b**) Force-displacement curve of Al-Cu and Al-Ni joints and (**c**) Stress-strain curve of Al-Cu and Al-Ni joints.

Figure 2 demonstrates static properties of Al-Cu and Al-Ni ultrasonic welding joints. USW welded samples using Ni and without Ni coating on Cu substrate are shown in Fig. 2(a). Force-displacement curve of Al-Cu and Al-Ni joints and corresponding displacements of elastic deformation zone, plastic deformation zone and failure zone are basically consistent. Results of this work indicated that under same welding parameters, Al line and T2 Cu and Ni-coated T2 Cu are basically the same. The ultrasonic welding joints of both terminals has the same strength. In working conditions that require ultrasonic welding of Al and Cu, nickel-coated copper can be used instead of Cu to prevent electrochemical corrosion or galvanic corrosion of Al and Cu. Figure 2(b) shows force-displacement curve of Al-Cu and Al-Ni joints where force and displacement are in direct proportion. The stress-strain relationship for a metal wire and metal sheet is shown in Fig. 2(c). Within proportionality limit, the Al-Cu and Al-Ni joints stress ∝ strain for the material of wire.

### Micromorphology and failure analysis

**Fig. 3:**
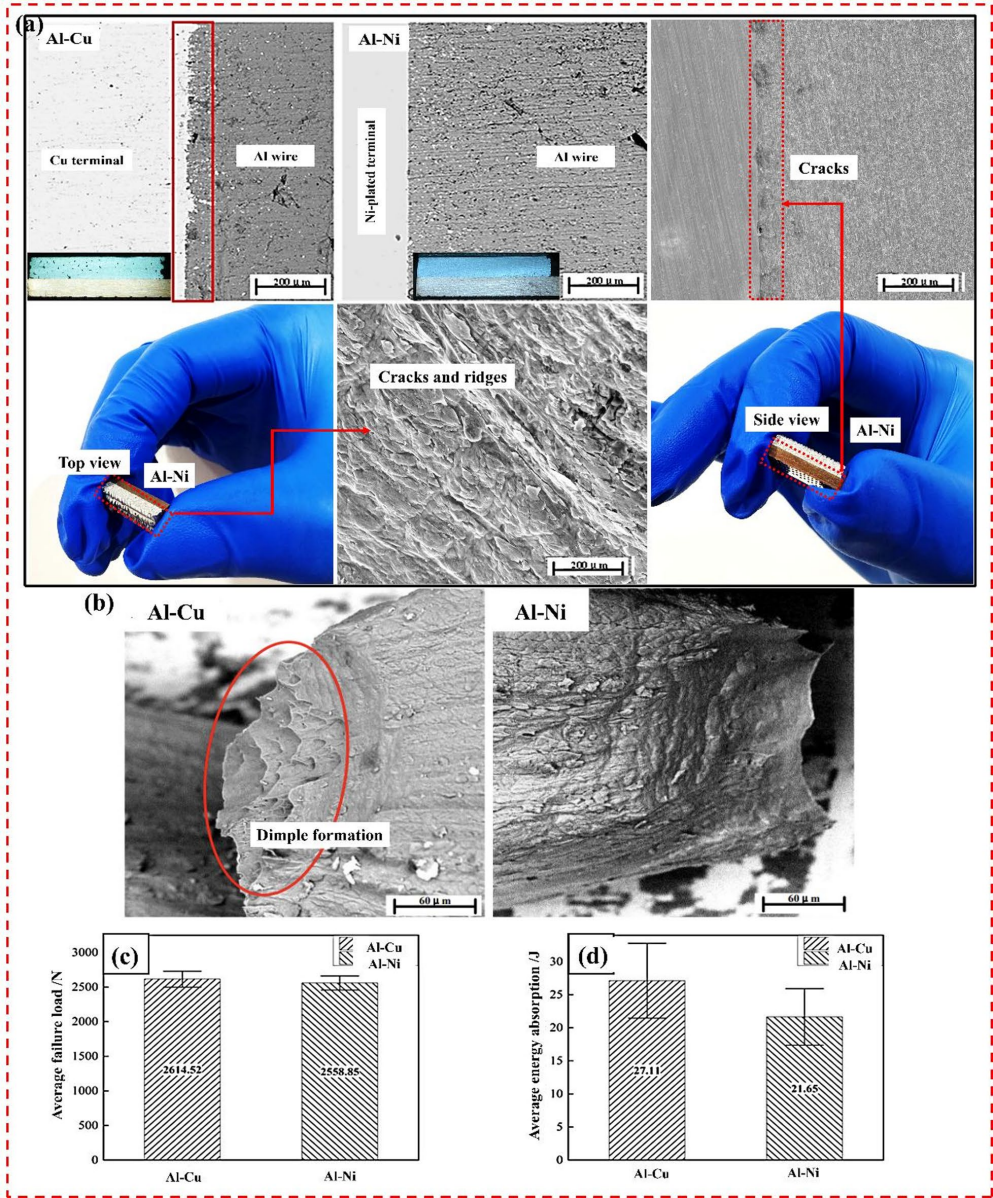
(**a**) Micromorphology analysis of joints using SEM (**b**) Failure analysis (**c**) Al-Cu, Al-Ni failure load change chart and (**d**) Energy absorption value change chart.

Figure 3a shows a cross-sectional SEM image of Al-Cu and Al-Ni welded joints. Micromorphological analysis of ultrasonically welded Al-Cu and Al-Ni joints was performed. Bond between wires within Al-Cu and Al-Ni connectors is relatively tight, but still has mechanical interlocking and a small number of pores. Copper at solder interface of Al-Cu connector diffuses significantly towards Al wire. Al-Ni joint soldering community shows large-area Cu diffusion does not occur on surface due to presence of cladding layer and only a wavy structure appears on terminal. Density and melting point of Ni are much higher than Cu and plastic flow is poor and both Ni and Cu are surface-centered cubic structure. Lattice constant of Ni is 0.3517 nm and Cu is 0.3608 nm. Without other internal stress interference, tensile stress forms in Ni layer and inhibits Cu diffusion. Figure 3b shows SEM images of Al-Cu and Cu-Ni joint failure. Wire breaks when the tensile force is greater than yield strength of welded wire, although both knots are caused by sharp necking of wire after elastic deformation. Eventually, this leads to joint failure. However, microscopic fracture morphology is very different for Al-Cu and Al-Ni joints. Al-Cu connector wire produces many dimples at fracture site, while no visible dimple structure is found in wire fracture of Al-Ni connector. So, energy absorption value of Al-Ni connector is lower than Al-Cu connector. This indicates that Al-Ni joint has weaker strength and low impact and seismic resistance under same welding parameters. Figure 3c,d displays static performance parameters of Al-Cu and Al-Ni joints. Average failure load of Al-Ni joint is 2558.85 N, which is 2.05% lower than average failure load of Al-Cu joint (2612.52 N). Average energy absorption of Al-Ni (21.65 J) joints is 20.14% lower than average capacity absorption of Al-Cu joints (27.11 J), indicating that coating has almost no effect on strength of welded joint, but will weaken the impact and seismic resistance of joint.

### Welded joint composition analysis

**Fig. 4:**
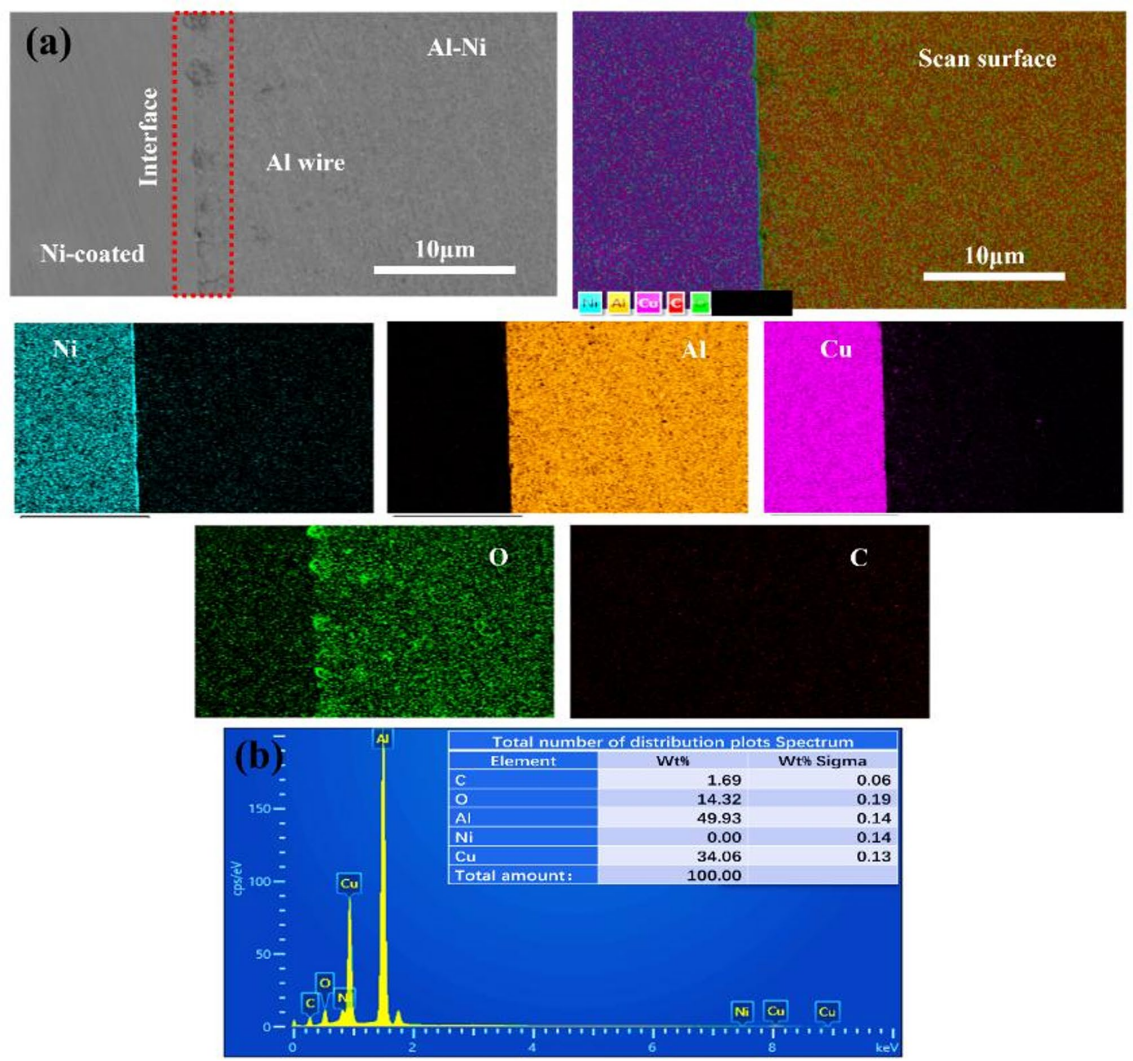
(**a**) The scanning electron microscope (SEM) analysis, energy dispersive X-ray spectroscopy (EDS) elements composition and elemental mapping for Al-Ni coated sample (**b**) Number of distribution plot spectrum.

The experimental conditions were controlled for consistency of results. The ambient temperature was maintained at 22 ± 2 °C with relative humidity at 50 ± 5% RH, minimizing their impact on the welding process and IMC formation^18^. Figure 4a illustrates composition analysis of Al-Ni joint using surface EDS scanning. Interface areas in Ni-coated sample were scanned to investigate metal diffusion phase and elemental composition in Al-Ni joint. Higher concentration of Al elements in joint is 49.93% under sigma weight of 0.14%. Concentration of Al on Cu surface shows a deep distribution of metals and increases strength and toughness of joint. Surface morphology of Al-Ni joint reduces formation of IMC due to production of less carbon content weighing 1.69% in Al-Ni. In EDS results, maximum carbon in Al-Cu joint was 10% which increases frictional energy during welding process. Certain oxides and impurities in Al-Ni will be reduced and persist in small amounts of oxides due to insertion of small wires within Ni-coated surface. High carbon content in welded joint often leads to formation of gases such as CO, which is difficult to remove from welded interface in later stage of crystallization and causes production of IMC. EDS investigates the surface performance caused by Ni coating in welds and reduces formation of carbon element leading to production of IMC which effects mechanical properties and overall performance of Al-Ni joints. The EDS spectrum for Al-Ni joint is shown in Fig. 4b.

## Conclusions

1. Nickel-coated copper (Cu-Ni) terminals have a minimal impact on the strength of the connector. However, the energy absorption capacity of Cu-Ni connector is 60.71% lower than Cu-Cu connector. The average energy absorption of Al-Ni joints (21.65 J) is 20.14% lower than Al-Cu joints (27.11 J). The buffering performance of welded joint between Ni-coated Cu terminal and two wire harness materials is reduced, indicating that Ni-coated Cu terminals cannot replace copper terminals in manufacturing applications. It requires high impact resistance and seismic performance.
2. Ni-coated Cu terminals also influence the formation of wire harness welding joints. The presence of coating prevents direct contact between Al wire and Cu structure of the terminal, hindering the plastic flow of material and inter-atomic bonding of base metal during ultrasonic welding. This inhibits metallurgical bonding to some extent, although the effect on joint strength is not significant.
3. The failure mode of Cu-Cu connector is fracture in welding area, suggesting that the strength of welding interface among wire and terminal is greater than the connection strength amid wires. In contrast, the failure mode of Cu-Ni connector is pull-off and indicates the welding strength between the connection strength and wires. Likewise, the failure mode of Cu-Ni connector is pull-off and demonstrates that the welding strength between the wires is higher than the strength amid wires. It is concluded that nickel-plated copper terminals can replace copper terminals in applications where high impact and seismic resistance are not required.
4. This study contributes to advancing the understanding of ultrasonic welding for this specific material combination and offers insights for improving the quality, efficiency and applicability of the process in various automotive and aerospace industries.

## Data Availability

The datasets used and/or analyzed during the current study are available from the corresponding author upon reasonable request.

## References

[1] Tsujino, J., Ihara, S., Harada, Y., Kasahara, K. & Sakamaki, N. Characteristics of coated copper wire specimens using high frequency ultrasonic complex vibration welding equipments. In *Ultrasonics* **42** 121–124 (2004). 10.1016/j.ultras.2004.01.051 15047272

[2] Luo, C. & Zhang, Y. Joining of copper foils via al/ni reactive multilayer nanofoils. *J. Mater. Process. Technol.* **298**, 117294 (2021).

[3] Abbas, Z., Zhao, L., Deng, J., Wang, S. & Hong, W. Advances in ultrasonic welding of lightweight alloys: A review. *High Temp. Mater. Process* **42** (2023).

[4] Lun, Z., Shicheng, W., Jiguang, L., Abbas, Z. & Gang, X. Performance enhancement of clinched joints with ultrasonic welding for similar and dissimilar sheet metals. *Weld. World*. 10.1007/s40194-023-01589-1 (2023).

[5] Ye, F., Lu, H. & Qi, H. Joints formation and bonding mechanism of ultrasonic welded multi-strand single core copper cables with copper terminals. *Mater. Lett.* **327**, 133015 (2022).

[6] Lu, H., Ye, F. & Wang, Y. Orthogonal experiments and bonding analysis of ultrasonic welded multi-strand single core copper cables. *J. Manuf. Process.* **78**, 1–10 (2022).

[7] Lee, D., Kannatey-Asibu, E. & Cai, W. Ultrasonic welding simulations for multiple layers of lithium-ion battery Tabs. *J. Manuf. Sci. Eng.* **135**, 1–14 (2013).

[8] Bagheri, B., Shamsipur, A., Abdollahzadeh, A. & Mirsalehi, S. E. Investigation of SiC nanoparticle size and distribution effects on microstructure and mechanical properties of al/sic/cu composite during the FSSW process: experimental and simulation. *Met. Mater. Int.* **29**, 1095–1112 (2023).

[9] Ni, Z., Zhao, H., Mi, P. & Ye, F. Microstructure and mechanical performances of ultrasonic spot welded al/cu joints with al 2219 alloy particle interlayer. *Mater. Des.* **92**, 779–786 (2016).

[10] Wang, K. et al. Unveiling non-equilibrium metallurgical phases in dissimilar Al-Cu joints processed by vaporizing foil actuator welding. *Mater. Des.* **186**, 108306 (2020).

[11] Rubino, F., Parmar, H., Esperto, V. & Carlone, P. Ultrasonic welding of magnesium alloys: a review. *Mater. Manuf. Process.* **35**, 1051–1068 (2020).

[12] Huang, H. et al. Heat generation and deformation in ultrasonic welding of magnesium alloy AZ31. *J. Mater. Process. Technol.* **272**, 125–136 (2019).

[13] Dhara, S. & Das, A. Impact of ultrasonic welding on multi-layered Al–Cu joint for electric vehicle battery applications: A layer-wise microstructural analysis. *Mater. Sci. Eng. A*. **791**, 139795 (2020).

[14] Ao, S. S. et al. Microstructure and mechanical properties of dissimilar NiTi and 304 stainless steel joints produced by ultrasonic welding. *Ultrasonics* **121** (2022). 10.1016/j.ultras.2022.106684 35033933

[15] Dak, G., Guguloth, K., Sirohi, S., Adin, M. Ş. & Pandey, C. Creep and High-Temperature tensile deformation behavior of the TIG welded P92/304L dissimilar steel weld joints. *J. Mater. Eng. Perform.*10.1007/s11665-024-09872-y (2024).

[16] Maurya, A. K. et al. Tribological performance of gas tungsten Arc welded dissimilar joint of sDSS 2507/IN-625 for marine application. *Arch. Civ. Mech. Eng.* **24**, 1–15 (2024).

[17] Zhao, D., Zhao, K., Ren, D. & Guo, X. Ultrasonic welding of Magnesium-Titanium dissimilar metals: A study on influences of welding parameters on mechanical property by experimentation and artificial neural network. *J. Manuf. Sci. Eng. Trans. ASME*. **139**, 1–9 (2017).

[18] Adin, M. Ş. A parametric study on the mechanical properties of MIG and TIG welded dissimilar steel joints. *J. Adhes. Sci. Technol.* **38**, 115–138 (2024).

